# Glycaemic potency reduction by coarse grain structure in breads is largely eliminated during normal ingestion

**DOI:** 10.1017/S000711452100252X

**Published:** 2022-05-28

**Authors:** Akila SRV, Suman Mishra, Allan Hardacre, Lara Matia-Merino, Kelvin Goh, Frederick J. Warren, John Alexander Monro

**Affiliations:** 1 The New Zealand Institute for Plant & Food Research Limited, Private Bag 11600, Palmerston North 4442, New Zealand; 2 Riddet Institute, Massey University, Private Bag 11 222, Palmerston North 4442, New Zealand; 3 Massey Institute of Food Science and Technology, Massey University, Private Bag 11 222 Palmerston North 4410, New Zealand; 4 Quadram Institute Bioscience, Norwich Research Park, Norwich NR4 7UA, Norfolk

**Keywords:** Glycaemia, Grain, Structure, Breads

## Abstract

The hypothesis that coarse grain particles in breads reduce glycaemic response only if the particles remain intact during ingestion was tested. Three breads were formulated: (1) White bread (WB – reference), (2) 75 % of kibbled purple wheat in 25 % white bread matrix (PB) and (3) a 1:1 mixture of 37·5 % kibbled soya beans and 37·5 % of kibble purple wheat in 25 % white bread matrix (SPB). Each bread was ingested in three forms: unchewed (U), as customarily consumed (C) and homogenised (H). Twelve participants ingested 40 g available carbohydrate portions of each bread in each form, with post-prandial blood glucose measured over 120 min. Glycaemic responses to WB were the same regardless of its form when ingested. Unchewed PB had significantly less glycaemic effect than WB, whereas the C and H forms were similar to WB. Based on a glycaemic index (GI) of 70 for WB, the GI values for the C, U and H breads, respectively, were WB: 70·0, 70 and 70, PB: 75, **42** and 61, SPB: 57, **48** and 55 (%) (Least significant difference = 17·43, *P* < 0·05, bold numbers significantly different from WB). The similar glycaemic response to the H and C forms of the breads, and their difference from the U form, showed that the glycaemia-moderating effect of grain structure on starch digestion was lost during customary ingestion of bread. We conclude that the kibbled-grain structure may not effectively retard starch digestion in breads as normally consumed because it is largely eliminated by ingestive processes including chewing.

Glucose intolerance, involving both post-prandial and chronic hyperglycaemia, is recognised as a global public health problem, associated with the metabolic syndrome, obesity, diabetes complex, and leading to multiple downstream medical complications^(1,2)^. The well-established damage caused by hyperglycaemia has led to a demand for foods formulated to reduce glycaemic impact.

One of the strategies used in the baking industry to reduce glycaemic impact is to include intact or partially intact grains, in products such as breads^(3,4)^. The assumption is that grain structure will impede digestive enzyme access to starch, reducing the rate of release of digestion products to the intestinal lumen, thus reducing the rate of glucose absorption and subsequent glycaemic response. However, initial oral and gastric processes associated with ingestion, including mastication, have evolved specifically to break down food structure and facilitate extraction of as much nutrient as possible from particulate foods such as grains. So, although particle size and intactness can have the clear effects of reducing starch digestion rate *in vitro* ^(5)^, inhibition of starch digestion is likely to be diminished by the processes of normal ingestion.

Ingestion is multifaceted. Chewing is a first step, when food is crushed while being moistened and mixed with saliva containing salivary amylase, as it is formed into a bolus^(6,7)^. Physical breakdown of food structure continues in the stomach while salivary amylase activity, which had initiated oral breakdown of starch, continues until reduced by low pH and pepsin activity in the gastric chyme^(8)^, as the bolus disintegrates. It has been estimated that as much as 59 % of starch in bread may be digested by the time food enters the small intestine^(9)^.

It is not only particle size that retards starch digestion but also the cellular structure within the particles. The starch in graminaceous (cereal) grains, such as wheat, is typically stored in endosperm tissue consisting of thin-walled starch-filled cells with the whole surrounded by a resistant pericarp. Once the pericarp is ruptured during ingestion, the starch is relatively accessible to digestive enzymes. In contrast, in legumes (pulses), the cell walls throughout the cotyledon tissue are robust, with a support function, and effectively encapsulate the starch within cells that restrict enzyme access until breached^(10)^. Therefore, the effectiveness of ingestive processes in making starch susceptible to digestion may differ between graminaceous and pulse grain particles.

The aim of the research reported here was to quantify the effects of normal ingestion on the glycaemic impact of foods that had been formulated on the basis of *in vitro* digestion to contain enough cereal and/or pulse grain structure to reduce their glycaemic impact *in vivo.* We hypothesised that the processes that are part of normal ingestion and assimilation – chewing, swallowing, multienzyme digestion, gastric shear and so on – will largely overcome the restrictions to starch digestion imposed by seed structure in kibbled-grains and pulses. Breads with a low rate of *in vitro* starch digestion due to their content of kibbled cereal or pulse grains were prepared, and the glycaemic response to them when swallowed without chewing was compared with the glycaemic response when they were ingested normally, or after homogenising to completely eliminate coarse structure. The study tested the inclusion of coarse cereal and pulse grain particles as a reformulation strategy for lowering glycaemic impact of breads, given that the human ingestion process is designed to overcome the restrictions that food structure places on digestion.

## Methods

### Test foods

The test foods consisted of three breads:

1. White bread (WB, control) – Bread with a 100 % white bread matrix and no kibbled grains.
2. Kibbled purple wheat (PB) – containing 75 % of kibbled (>2·8 mm) purple wheat and 25 % of white bread matrix.
3. Kibbled soya-purple wheat (SPB) – containing 37·5 % kibbled (>2·8 mm) soya, 37·5 % kibbled (>2·8 mm) purple wheat and 25 % of white bread matrix.

The kibbled soya and purple wheat grains had been prepared by passing the whole grains through a Kenwood grain mill (AT941A) with fluted rollers adjusted to give the desired particle size spectrum, and the particles winnowed with an air blower and finally sieved using a mechanical shaker with a 2·8 mm screen (Model RX-6-1, W.S Tyler, 8570 Tyler Blvd.) to obtain the large kibble (>2·8 mm) required for the experimental breads. The grain particles were soaked overnight and blotted dry before breadmaking. The formulation of the breads is given in Table 1 and full details of the bread preparation in Supplementary material.

**Table 1.** Ingredients and formulations for white (WB) and kibbled-grain breads (g)

Ingredients (g)	WB	75 % PB	75 % SPB
Flour	100	100	100
Sugar	1·89	1·89	1·89
Salt	1·89	1·89	1·89
Gluten	3·77	3·77	3·77
Yeast	3·21	3·21	3·21
Dough improver	0·57	0·57	0·57
Oil	3·40	3·40	3·40
Water	73·96	73·96	73·96
Kibbled-grains	N/A	300 purple wheat*	150 soya; 150 purple wheat*

PB, purple kibbled wheat bread; SPB, kibbled soya/kibbled purple wheat bread.

* Dry weight, but grains prehydrated prior to breadmaking.

### Digestive analysis of the test foods

Each of the test breads was subjected to an *in vitro* digestive analysis in both the intact and homogenised forms, to determine the relative release of glucose equivalents with time, and the total potentially available carbohydrate, where ‘potentially available’ means digested if unoccluded by particle mass, in each of the breads. Therefore, it includes the Type 1 resistant starch that would have been present in the coarse particles due to grain structure. An *in vitro* digestion procedure, using 5 g of the bread in a volume of 50 ml of digest, was used^(11)^. The samples were moistened and either gently crumbled or homogenised in 30 ml of deionised water before adjusting to pH 2·5 with 1 M hydrochloric acid. A volume of 1 ml of 10 % pepsin (Sigma P-7125) solution in 0·05 M hydrochloric acid was added, and the pots incubated at 37°C for 30 min to simulate gastric digestion. The pH was then adjusted to 6·5 with 0·1 M sodium hydroxide (NaOH) and maleate buffer (5 ml, 0·2 M, pH 6·5) and made accurately to 50 ml with deionised water, followed by addition of pancreatin (Sigma P-7545) solution (5 % w/v, 0·2 ml) and amyloglucosidase (Megazyme E-AMG, 0·1 ml). The pancreatic digestion was continued for 120 min with sampling (1·0 ml) at intervals of 0, 10, 20, 30, 40, 60 and 120 min. The 120 min sample of the homogenised breads was used to determine the content of potentially available carbohydrate in each bread. The 1 ml samples of digesta from each sampling time point were added to 4 ml ethanol and mixed. After centrifuging, an aliquot of the ethanolic supernatant was subjected to an amyloglucosidase–invertase secondary digestion to convert maltose and limit dextrins to glucose. Free sugar was measured spectrophotometrically as glucose equivalents using the dinitrosalicylic acid method^(12)^. As starch was the only digestion product, no allowance was necessary for non-glucose sugars, so the glucose equivalents represented glycaemic glucose equivalents (GGE), defined as the amount of glucose of the same glycaemic load as a given carbohydrate source^(13)^.

Digestograms of the release of glucose with time of digestion were plotted. Theoretical glucose disposal lines derived from clinical trials^(11)^ were inserted, and the difference between glucose disposal and glucose release was calculated. The resulting curves of net GGE accumulation with time gave simulated blood glucose response curves, as previously described^(11)^. The AUC were determined by the trapezoid summation method routinely used in glycaemic index (GI) determination. By comparing the area under the net GGE curve of a bread with the area for homogenised white bread, relative glycaemic potency (RGP) values were obtained for comparison with those calculated from clinical blood glucose responses. This comparison gave an indication of the correspondence between *in vitro* and *in vivo* starch digestion in support of basing the clinical trial on *in vitro* digestibility data.

### Formulation of meals

The nine test meals were each formulated to contain 40 g of potentially available carbohydrate, based on the *in vitro* digestive analysis of the available carbohydrate content of homogenised samples (WB, 38·1 %; PB, 31·7 %; SPB 24·6 % available carbohydrate), and on moisture contents of the breads measured according to an official AOAC method^(14)^ (WB, 40·2 %; PB, 47·2 % and SPB, 49·1 %). The breads were baked weekly, divided into the 40 g available carbohydrate portions for ingestion and frozen. Prior to ingesting, the breads were thawed to room temperature overnight (14 h). Starch digestibility was determined to confirm that freezing, storing and thawing the breads had not altered the digestibility of the starch. Breads for treatment H were homogenised with 150 ml of water not more than 1 h before being consumed. Based on the analyses conducted, all meals contained the same mass of potentially digestible carbohydrate and water (Table 1).

### Trial procedure

**Fig. 1.**
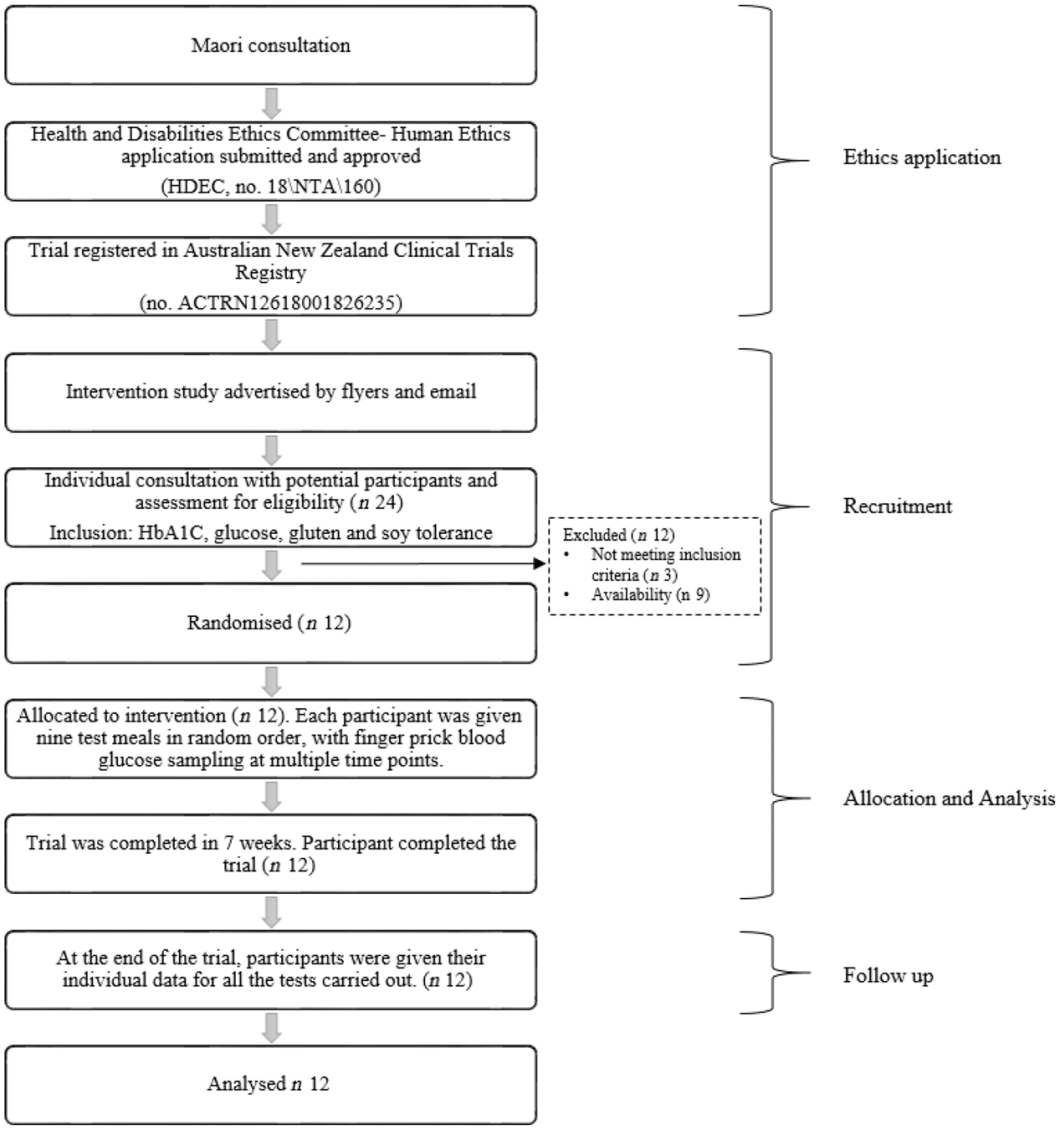
Human intervention study flow chart showing ethical approval, recruitment and intervention processes for this trial. HbA1c, glycated Hb.

The clinical trial was carried out in the New Zealand Institute for Plant and Food Research clinical suite. Ethical approval was obtained from the New Zealand Health and Disabilities Ethics Committee (HDEC, no. 18\NTA\160), and the trial was registered with the Australia New Zealand Clinical Trials Registry (no. ACTRN12618001826235). The participant flow chart is given in Fig. 1 and the CONSORT checklist in Supplementary material.

Twenty-four volunteers were recruited for the initial screening using flyers or emails that briefly described the study. Volunteers were pre-screened and asked initial recruitment questions to determine their suitability to participate in the study, and the nature of the study and their involvement and responsibilities described. Eligible volunteers willing to participate in the study were presented with an information sheet containing study details and an informed consent form. Their fasting blood glucose concentration and glycated Hb (HbA1c) were measured to ensure that they were within the normal (non-diabetic) range and to familiarise them with the blood sampling procedure to be used.

A total of twelve participants (five male and seven female) were selected as suitable for the final study. The characteristics (mean values and standard deviations) of the study group were: age 33·3 (sd 11·7) years, BMI 23·6 (sd 3·3) kg/m^2^, fasting glucose 4·4 (sd 0·3) mmol/l and HbA1c 34·5 (sd 4·5) mmol/mol. The participant number (*n* 12) exceeded the minimum (*n* 10) specified by the current ISO method (ISO 26642:2010) for determining GI and was typical of studies comparing foods. The twelve participants were from within Plant and Food Research and Massey University at Palmerston North and satisfied the following exclusion criteria:

- Age: Below 18 or above 65 years.
- BMI: BMI below 18 and above 35 kg/m^2^.
- Glucose intolerance: History of diabetes or evidence of glucose intolerance in a preliminary test.
- Gluten and soya intolerance: History of intolerance to gluten, soya or bread products.
- Non-fasting: Unwilling to not consume anything apart from water in the 12 h before the test.
- Recent ill health.

A non-blinded randomised repeated-measures design was used in which the order of the treatments was randomised by computer for each participant and each participant ingested each of nine treatments once. The treatments were the three breads – white bread (WB), kibbled purple wheat bread (PB) and soya/kibbled purple wheat bread – each ingested in three forms – as was customary for the participant (‘chewed’) (C), unchewed (U) and homogenised (H) (Table 1). It was not practicable to blind the subjects to the breads they were ingesting, but the data analysis was completed by an investigator who was blinded to the identity of samples and treatments.

### Ingestion of test foods

Participants were asked to attend on weekday mornings. In preparation for each testing session, participants were requested to:

- Avoid strenuous physical activity, smoking or consuming alcohol the evening before and the day of a test.
- Consume a similar carbohydrate-based meal the evening before each test.
- Fast from 21.00 hours the night before a test, with water consumption not restricted.
- Allow at least 48 h (wash-out time) between tests.

On each test day, the volunteers were seated and asked to remain so for the duration of the test. Once each subject was relaxed and comfortable for 10 min, a baseline blood sugar measurement was taken in duplicate. Each subject was then given a test food and instructed to consume the whole amount within 10 min. The meals included enough water to enable swallowing without chewing in the unchewed treatments (Table 2). Blood glucose was measured in finger prick blood samples collected at 0 (baseline), 15, 30, 45, 60, 90 and 120 min using a HemoCue® blood glucose meter.

**Table 2.** Composition of test meals to ensure the same intake (40 g) of available carbohydrate and water in each meal

Test foods	Treatment	Weight of bread (g)	Water for drinking (ml)	Water for homogenising (ml)
WB	C	105	338·2	
	U	105	338·2	
	H	105	188·2	150
PB	C	126	321	
	U	126	321	
	H	126	171	150
SPB	C	163	300	
	U	163	300	
	H	163	150	150

WB, white bread; PB, purple kibbled wheat bread; SPB, kibbled soya/kibbled purple wheat bread; C, Chewed; U, unchewed; H, homogenised.

### Analysis of glycaemic response data

All twelve of the subjects completed the trial, and no obvious outliers were detected, and all results were included in the analysis. The blood glucose concentration changes from baseline were plotted against time to obtain blood glucose response curves. Each individual’s baseline fasted blood glucose value was subtracted from subsequent measurements to obtain the incremental blood glucose response from which the incremental area under the blood glucose response curve (iAUC) was derived by trapezoid summation. The highest post-prandial blood glucose peak for each individual, irrespective of the time of occurrence (nearly all were at either 30 or 45 min), was used to determine the mean peak height for each meal.

### Glycaemic index

As all bread meals had been formulated to supply the same (40 g) available carbohydrate, the GI value for each treatment could be estimated from its iAUC value up to 120 min, relative to that of WB, which was used as the reference with an assumed GI of 70:

GI_food_ = 70 × iAUC_food_/iAUC_WB._

### Relative glycaemic potency

RGP of the breads refers to the blood glucose-raising effect of ingesting 100 g whole bread relative to the effect of ingesting 100 g of glucose, expressed as grams of glucose equivalents (GGE). As a GI of 70 % for available carbohydrate in white bread (WB) means that the carbohydrate has a relative glycaemic impact that is 70 % that of glucose, or 70 GGE/100 g available carbohydrate, the RGP of any other food ingested at the same available carbohydrate intake may be calculated from the glycaemic response to the food carbohydrate relative to white bread carbohydrate (iAUC_food_/iAUC_WB_), adjusted by the percentage of available carbohydrate in the bread.

RGP_food_ = %CHO_food_/100 × iAUC_food_/iAUC_WB_ × 70

Data were entered into a Microsoft® Excel spreadsheet for preliminary analysis. For statistical comparison of means (ANOVA), GenStat software was used (version 11.1; VSNi Ltd). Data were analysed using ANOVA blocked by individual, testing differences between foods and treatments. Statistical analysis described the differences between the foods in their effects on blood glucose concentrations at different post-prandial time points and allowed the precision of the glycaemic potency values to be determined. *P* values ≤ 0·05 were considered significant.

## Results

### *In vitro* digestive analysis

**Fig. 2.**
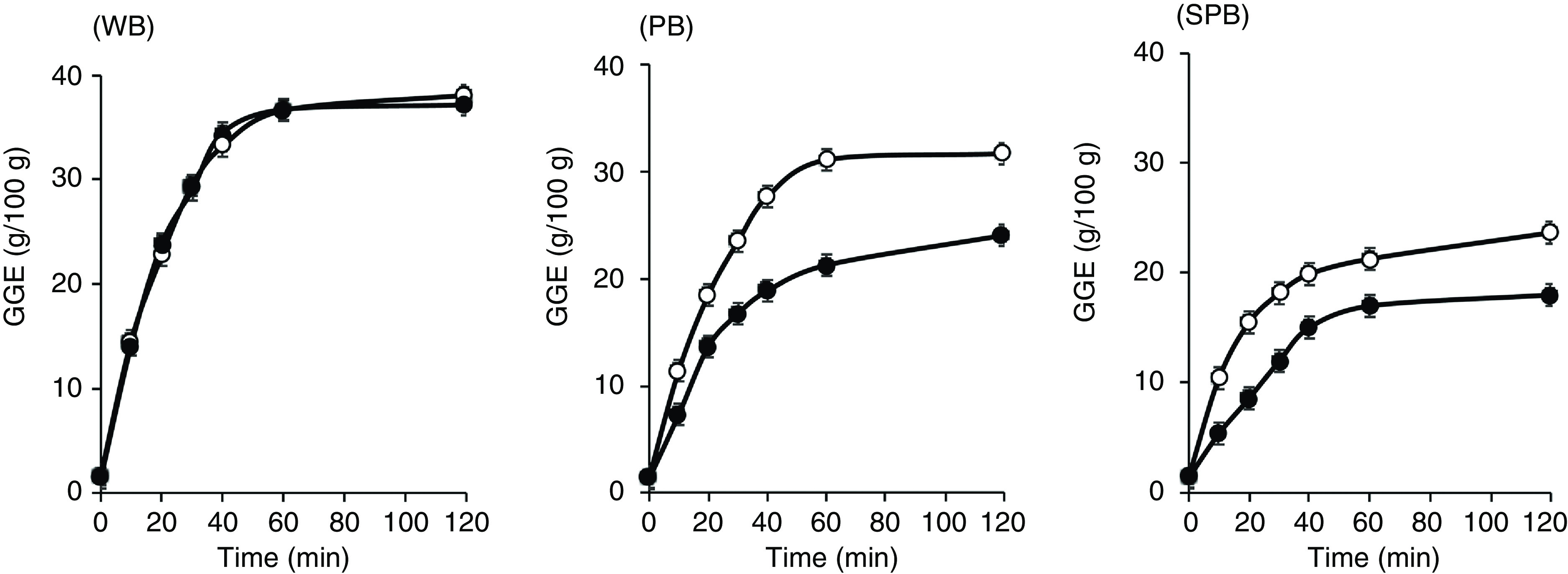
Sugar release as glucose equivalents (GE)/100 g bread during *in vitro* digestion of white bread (WB), purple wheat bread (PB) and soya/purple wheat bread (SPB), digested intact (&x25CF;) or homogenised (○). The average precision of measurements was sd = 0·5 g and the CV 1·75 %.

The *in vitro* digestive analysis revealed large differences between the breads (Fig. 2). Homogenising to eliminate coarse grain structure had no effect on the rate or extent of white bread digestion. In contrast, the extent and rate of digestion of the kibbled-grain breads were much lower for the intact than for the homogenised forms, and digestion of the kibbled-grain breads tended to plateau well before digestion was complete. After 120 min, the digestion of intact WB was 98 % of homogenised WB, whereas the digestion of intact PB and SPB breads was both 76 % of the homogenised sample and remained at 76 % after further digestion to 180 min (not shown).

Mean values for glucose equivalent release from the homogenised sample at 120 min, used as ‘available carbohydrate’, were 38·1 g/100 g for WB, 31·7 g/100 g for PB and 23·7 g/100 g for SPB.

RGP values calculated from the net AUC for glucose equivalent release during digestion *in vitro* (Fig. 2), after subtracting theoretical blood glucose disposal, were (where I = intact and H = homogenised): WBI, 25·5; WBH, 26·6; PBI, 14·7; PBH, 21·4; SPBI, 10·4; SPBH 16·5 g glucose equivalents/100 g bread. The *in vitro* data therefore predicted a much higher glycaemic impact per given mass of bread for the white bread than for the kibbled breads.

### Glycaemic response

Both blood glucose response amplitude and iAUC differed significantly between breads and treatments (Table 3) despite large between-subject variations in the blood glucose responses typical of such studies.

**Table 3.** Characteristics of the blood glucose response curves during 0–120 min after ingestion of three breads in three forms, and glycaemic indexes and relative glycaemic potency values derived from the blood glucose response data (Mean values with their standard errors)

	WB			PB			SPB						
	C	U	H	C	U	H	C	U	H	LSD (*P* < 0·05)	Bread F (2, 88 df)	CUH F (2, 88 df)	Bread × CUH F (4, 88 df)
Amplitude (mmol/l)													
Mean	2·61^abc^	2·76^abc^	3·03^ab^	3·24^a^	1·94^d^	2·72^abc^	2·73^abc^	2·23^bc^	2·41^bcd^	0·67	1·6	4·3	2·6
sem	0·20	0·23	0·29	0·42	0·21	0·31	0·26	0·31	0·24			*	*
%WB(C)	100·0	106	116	124	74	104	105	85	92	26			
iAUC (mmol/l × min)													
Mean	182^ab^	173^ab^	176^ab^	194^a^	100^d^	152^abc^	160^abc^	125^cd^	140^bcd^	48	3·6	5·4	1·7
sem	13·3	18·2	24·7	26·9	12·8	22·9	16·5	18·3	15		*	**	
%WB(C)	100	95	97	107	55	83	88	69	77	26			
GI													
Mean	70·0^ab^	69·5^ab^	70·4^ab^	76·7^a^	41·5^c^	60·9^abc^	63·0^abc^	49·0^bc^	55·3^abc^	18	4·1	5·2	1·9
sem	0·0	7·13	9·78	10·05	6·49	8·78	5·71	7·12	5·55		*	**	
%WB(C)	100	99	101	110	59	87	90	70	79	26			
RGP (GGE g/100 g bread)													
Mean	26·7^a^	26·5^a^	26·8^a^	23·8^ab^	13·2^cd^	19·3^bc^	14·0^cd^	11·7^d^	13·6^cd^	5·7	33·5	3·6	1·8
sem	0·00	2·72	3·73	3·08	1·97	2·65	1·03	1·69	1·28		***	*	
%WB(C)	100	99	101	89	50	72	53	44	51	21			

WB, White bread; PB, kibbled purple wheat bread; SPB, kibbled soya/purple wheat bread; C, chewed; U, unchewed; H, homogenised; GI, glycaemic index based on response to 40 g available carbohydrate in all treatments with the normally consumed white bread (WB(C), GI = 70) used as the reference; %WB (C), % of (WB(C) value. Values in a row with a common letter in the data label do not differ significantly (*P* < 0·05 based on least significant difference); iAUC, incremental area under the blood glucose response curve; GGE = glycaemic glucose equivalents (g). * *P* < 0·05, ** *P* < 0·01, *** *P* < 0·001.

#### Response amplitude (peak height)

**Fig. 3.**
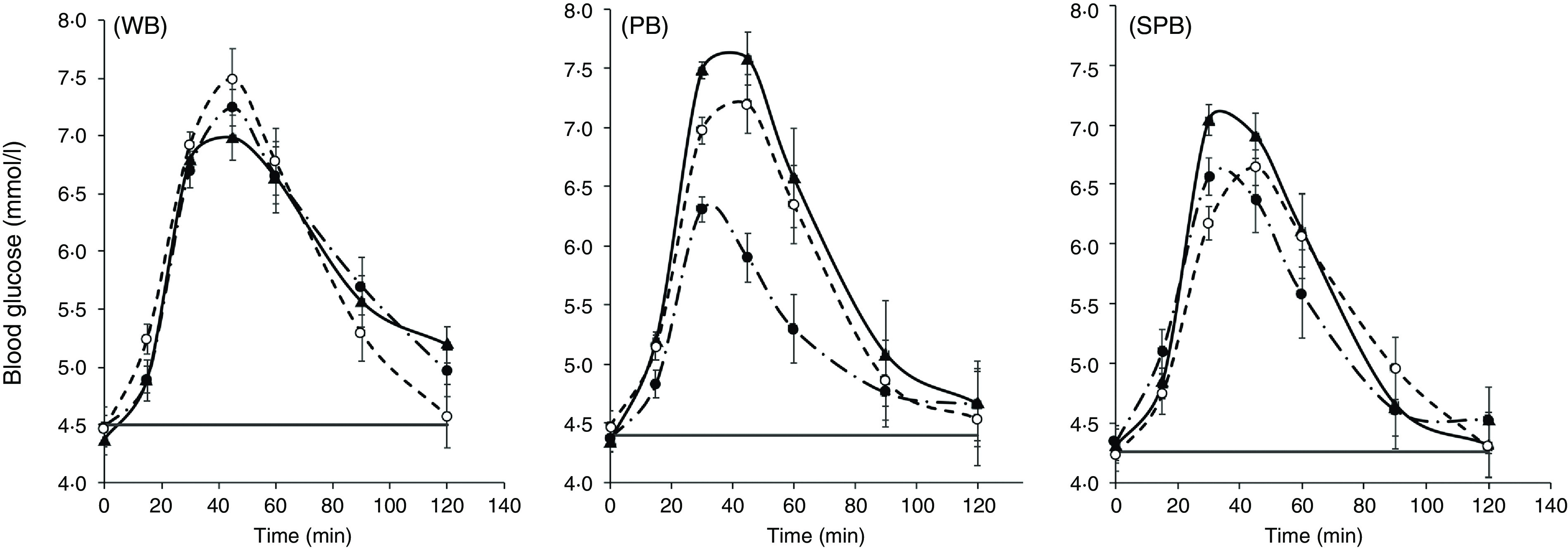
Blood glucose responses to 40 g carbohydrate intakes of white bread (WB), purple wheat bread (PB) and soya/purple wheat bread (SPB) each ingested chew©(C), unchewed (U) or homogenised (H).

Plasma glucose concentrations reached peak values between 30 and 45 min after the ingestion of test breads and decreased thereafter as a result of metabolic glucose disposal (Fig. 3). The mean peak values (mmol/l) for most treatments, except PB(U) (1·94 (sd 0·21)) and PB(C) (3·24 (sd 0·42)), were similar and not significantly different, falling with the range 2·41–3·03 (mean 2·71, sd 0·20). PB(U) was significantly (*P* < 0·05) lower than PB(C), and SPB(U) was lower than SPB(C), but not significantly so (*P* < 0·05). Peak heights for the three forms in which WB was ingested (C, U, H) were very similar to one another (Table 3).

For both the PB and SPB breads, chewing and homogenisation resulted in a peak amplitude similar to that of the WB. However, swallowing PB and SPB without chewing caused a substantial and statistically significant reduction in the peak amplitudes of about 26 % and 15 %, respectively, of the WB treatments (Fig. 2 and Table 3).

#### The area under the blood glucose response curve

The mean iAUC for all ingested forms (C, U, H) of the white bread (WB) – chewed, unchewed and homogenised were similar (range: 173·4–182·1 mmol/l per min) differing from one another by <5 % (Table 3). The purple wheat bread (PB) when ingested chewed (C) had a mean iAUC (194·5 mmol/l) which was also similar to that of WB, but the iAUC was 17 % less than that of the WB when it was ingested homogenised, and 45 % less when ingested unchewed. The C and H forms of the soya/purple wheat bread (SPB) also induced lower iAUC than WB by 12 % and 23 %, respectively (Table 3). The greatest mean reductions in iAUC compared with white bread occurred upon ingestion of the unchewed (U) forms of PB (45 % and statistically significant, *P* < 0·05) and SPB (31 %).

#### Glycaemic index

The GI values for the WB samples based on a reference value of 70 for the customarily ingested (therefore chewed) WB sample C were unaffected by physical form in which they were ingested.

The unchewed PB and SPB breads had GI values of <55 so would be classified as ‘low GI’, while the homogenised PB and SPB samples both fell into the ‘medium GI’ (55–69) category. GI was significantly lower than WB only for the unchewed PB(U) (–41 %) and SPB(U) (–30 %) samples, reflecting the protection of starch from digestion by kibbled-grain structure^(15)^. The chewed PB had the highest GI value.

#### Relative glycaemic potency

While GI refers to the glycaemic impact of food available carbohydrate and is expressed on a carbohydrate only basis, RGP refers to the RGP of the whole food. The differences in RGP of the breads (Table 3) were more striking than the differences for other variables. All forms of the granular breads (PB and SPB) were substantially lower in glycaemic impact than the white bread, and all except the PB(C) significantly so (*P* < 0·05). The unchewed forms of PB and SPB had the lowest glycaemic impact. The glycaemic impact of the white bread was the same in all three forms.

**Fig. 4.**
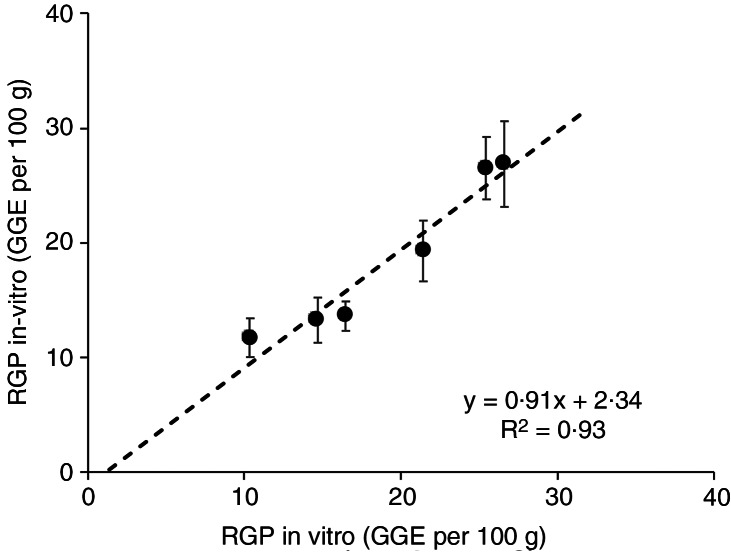
Correspondence between relative glycaemic potency determined *in vitro* and *in vivo* for homogenised and intact white and kibbled-grain breads.

The clinically determined relative glycaemic potencies of the unchewed breads PB(U) and SPB(U) were 69 % and 86 %, respectively, (average 77 %) of the RGP values for the homogenised forms (PB(H)) and SPB(H). Differences between intact (I) and homogenised (H) forms *in vitro* were 69 % for PB and 63 % for SPB. The clinically determined RGP values for unchewed and homogenised breads (Table 3) correlated closely (*R*2 = 0·93) with the RGP values based on *in vitro* digestion and adjusted for theoretical blood glucose disposal (Fig. 4), as described in the methods.

## Discussion

The close correspondence between the RGP values determined by *in vitro* digestion and those derived from true blood glucose responses to the breads in this study suggests that *in vitro* digestion can give an accurate indication of the impact of starch digestibility, as affected by food structure, on blood glucose responses. At the same time, because of the large *in vivo* differences in glycaemic impact between chewed and unchewed forms of the same breads (Table 3), the sensitivity of the *in vitro* method to physical structure suggests a need to account for the physical effects of normal ingestion, when assessing the benefit of grain particles, in reformulating for reduced glycaemic impact.

The hypothesis that retardation of starch digestion by coarse grain structure in breads would be largely removed by the processes of customary ingestion was confirmed. In the intervention study, all treatments contained the same quantity of potentially available carbohydrate, allowing comparison with the white bread, which was free of coarse kernel structure. For both the kibbled wheat (PB) and the kibbled soya/kibbled wheat (SPB) breads, the customarily consumed sample induced a blood glucose response amplitude and an iAUC that was not significantly or sizeably less than that of white bread. In contrast, the samples in which the grain kibbles were swallowed intact (samples PB(U) and SPB(U)) gave the lowest peak heights and AUC, while the white bread, which had no grain structure, was unaffected by the way in which it was ingested, consistent with the *in vitro* findings (Fig. 2). Therefore, the limitations to starch digestion that were imposed by kibble structure, and exposed by *in vitro* digestion, had been eliminated by normal ingestive processes.

The finding that the C, U and H samples of WB were very similar in glycaemic response amplitude, iAUC and GI may reflect the fact that the white bread lacked any structural impediment to starch digestion that could be removed by homogenising or chewing^(16)^. Assuming that bolus formation would occur with the chewed form of white bread, the results suggest that the bolus structure had little effect on glycaemic response, perhaps because of a lack of components, such as polysaccharides and remnants of structural elements present in whole kernels, that might prolong bolus cohesion and thus amylase activity within it.

The GI (GGE per 100 g of carbohydrates) for the breads followed the same trends reported for peak amplitude and iAUC (Table 3). For the homogenised forms of the PB and SPB breads peak amplitude and iAUC, and therefore GI was less than that for the chewed samples. Although the difference was not statistically significant, it provides circumstantial evidence that homogenisation was less effective in preparing particulate foods for starch digestion than chewing. That normal oral processing leads to the highest glycaemic impact in the whole grain breads is reasonable, since human dentition and mastication has evolved specifically to reduce food structure and convert potentially digestible food components to available nutrients.

A number of elements of the normal ingestion process that are missing when homogenised sample is ingested may explain why the homogenised samples did not have a higher glycaemic impact than the corresponding normally ingested (C) samples. They include crushing and mixing with more salivary amylase over a much greater time period than it would take to simply swallow the homogenised sample, and formation of a bolus. Bolus formation involving crushing and physical confinement within the protected amylolytic environment of the bolus has been shown to be important for the oral digestion process^(6)^ and is likely to be affected by the texture of the bread. The homogenised slurry could disperse more quickly than a bolus in the acidic gastric phase, reducing the possibility of salivary amylase activity persisting within the bolus or fragments of it^(8)^. Crushing and mixing with salivary amylase during mastication appear to be a precondition for rapid starch availability and make an important contribution to digestion of starches in the immediate post-prandial period of acute glycaemic response^(9)^.

The sample in which ingesting bread intact gave the greatest depression of glycaemic response was PB, whereas one might expect the intact SPB bread to give the lowest response because of the reported resilience of legume cotyledon cells encapsulating starch^(10)^. It appears that the occlusive effect of a solid mass of gelatinised starch in intact cooked endosperm may have retarded digestive enzyme access more than cell walls in cotyledon fragments, in which the starch is separated into individual cell contents, and protein digestion coupled with cell wall changes may have facilitated digestive enzyme access to the starch^(17,18)^. It is also possible that the texture imparted by soya bean grains in the SPB bread may have induced more crushing and more salivary amylase incorporation into SPB boli than into PB boli, reducing the difference in starch digestion between the U and H forms of the SPB bread. Further research, in which chewing characteristics are measured, could help resolve this question.

The glycaemic response data (peak height, iAUC and GI in Table 3) suggest that comparing kibbled-grain and white breads in equal carbohydrate portions may not reveal significant differences in glycaemic potency that exist between breads consumed in customary portions, or servings. When equal weights of the breads were consumed, the differences in glycaemic impact between the white and kibbled-grain breads were quite substantial and statistically significant because in equal bread weights carbohydrate intakes differed. For instance, the *in vitro* analysis showed the SPB bread, which contained high proportions of soya and purple wheat >2·8 mm, had a carbohydrate content of 24·6 % compared with a much higher available carbohydrate content of 38·1 % for WB. In all forms (chewed, unchewed and homogenised), SPB bread had a low RGP compared with white bread (WB).

The results indicate why GI, which is based on equal carbohydrate comparisons rather than customarily consumed portions, can be quite misleading as a guide to the relative glycaemic impact of food portions at point of sale. People eat foods and not only the carbohydrates in them. Glycaemic impact expressed as glycaemic glucose equivalents per weight of food may be a more understandable and practical estimate of glycaemic effect because it may be based on familiar or customarily consumed quantities, such as 100 g or a serving of bread.

Reducing the proportion of glycaemic material in the bread formulation could be a more effective and reliable strategy to decrease the glycaemic potency of breads than inclusion of whole grains alone and could be a focus of future research for the development of breads for reduced glycaemic potency. However, it is important to ensure that any apparent gain that results from reformulation does not have negative outcomes, such as has been suggested when fructose (GI = 19) is substituted for glucose (GI = 100)^(19)^.

In the present study, all participants consumed all breads and the focus was on comparison of breads rather than of individuals. However, it is worth noting that the degree to which ingestion alters starch digestibility is likely to differ between individuals and depend on their characteristic eating behaviours. Fast eaters have been reported to exhibit a lower post-prandial glycaemic response than slow eaters^(20,21)^. Differences in bolus particle size at point of swallowing, and effects of bolus properties on amylase activity and glucose release also depend on individual chewing characteristics^(20,22)^. Furthermore, large individual differences in human salivary amylase activity associated with differences in human salivary amylase gene copy number have been shown^(23)^. Thus, effects of coarse grain particles in breads that were not large in the present study may emerge more strongly in a comparison of participants differing in chewing characteristics and/or human salivary amylase activity.

### Conclusion

Kernel structure may play a significant role in reducing the digestibility of starch in bread, but this translates to a commensurate reduction in glycaemic potency only if the effects of grain structure survive the ingestion process. The results presented here suggest that grain structure will reduce glycaemic response if effects of ingestion, such as crushing, can be minimised. Very coarse grain particles may not, therefore, be most suitable in reformulating for reduced glycaemic impact if they induce a mastication response. The results also showed the importance of available carbohydrate content per customarily consumed portion in determining glycaemic impact. Reformulating breads for reduced glycaemic impact therefore needs to focus on substituting highly glycaemic components as well as minimising disintegration of starch-containing particles during ingestion, while maintaining organoleptic attributes.

## Supplementary material

For supplementary material accompanying this paper visit https://doi.org/10.1017/S000711452100252X. click here to view supplementary material

## References

[1] Monnier L & Colette C (2015) Postprandial and basal hyperglycaemia in type 2 diabetes: contributions to overall glucose exposure and diabetic complications. Diabetes Metab 41, S9–S15. 10.1016/S1262-3636(16)30003-9 26774019

[2] Brownlee M (2001) Biochemistry and molecular cell biology of diabetic complications. Nature 414, 813–820. 11742414 10.1038/414813a

[3] Jenkins DJA , Wesson V , Wolever TMS , et al. (1988) Wholemeal versus wholegrain breads – proportion of whole or cracked grain and the glycemic response. Br Med J 297, 958–960. 3142566 10.1136/bmj.297.6654.958 PMC1834634

[4] Liljeberg H , Granfeldt Y & Bjorck I (1994) Metabolic responses to starch in bread containing intact kernels *v.* milled flour. Am J Clin Nutr 59, 779S–779S. 1396475

[5] Akila SRV , Mishra S , Hardacre A , et al. (2019) Kernel structure in breads reduces *in vitro* starch digestion rate and estimated glycaemic potency only at high grain inclusion rates. Food Struct 21, 100109.

[6] Bornhorst GM & Singh RP (2012) Bolus formation and disintegration during digestion of food carbohydrates. Compr Rev Food Sci Food Saf 11, 101–118.

[7] Gao J , Wong JX , Lim JC-S , et al. (2015) Influence of bread structure on human oral processing. J Food Eng 167, 147–155.

[8] Freitas D & Le Feunteun S (2018) Acid induced reduction of the glycaemic response to starch-rich foods: the saliva–y -amylase inhibition hypothesis. Food Funct 9, 5096–5102. 30230497 10.1039/c8fo01489b

[9] Freitas D , Le Feunteun S , Panouille M , et al. (2018) The important role of saliva–y -amylase in the gastric digestion of wheat bread starch. Food Funct 9, 200–208. 29260815 10.1039/c7fo01484h

[10] Do DT , Singh J , Oey I , et al. (2019) Modulating effect of cotyledon cell microstructure on *in vitro* digestion of starch in legumes. Food Hydrocolloids 96, 112–122.

[11] Monro JA , Mishra S & Venn B (2010) Baselines representing blood glucose clearance improve *in vitro* prediction of the glycaemic impact of customarily consumed food quantities. Br J Nutr 103, 295–305. 19930760 10.1017/S0007114509991632

[12] Englyst HN & Hudson GJ (1987) Colorimetric method for routine analysis of dietary fibre as non-starch polysaccharides. A comparison with gas liquid chromatography. Food Chem 24, 63–76.

[13] Monro JA & Shaw M (2008) Glycemic impact, glycemic glucose equivalents, glycemic index, and glycemic load: definitions, distinctions and implications. Am J Clin Nutr 87, 237S–243S. 18175763 10.1093/ajcn/87.1.237S

[14] I. AOAC (1995) Official Methods of Analysis of AOAC International. Arlington, VA: AOAC Intl pv (loose-leaf).

[15] Edwards CH , Grundy MM , Grassby T , et al. (2015) Manipulation of starch bioaccessibility in wheat endosperm to regulate starch digestion, postprandial glycemia, insulinemia, and gut hormone responses: a randomized controlled trial in healthy ileostomy participants. Am J Clin Nutr 102, 791–800. 26333512 10.3945/ajcn.114.106203 PMC4588739

[16] Burton PM , Monro JA , Alvarez L , et al. (2011) Glycemic impact and health: new horizons in white bread formulations. Crit Rev Food Sci Nutr 51, 965–982. 21955095 10.1080/10408398.2010.491584

[17] Pallares AP , Miranda BA , Truong NQA , et al. (2018) Process-induced cell wall permeability modulates the in vitro starch digestion kinetics of common bean cotyledon cells. Food & Funct 9, 6545–6555. 10.1039/c8fo01619d 30480698

[18] Rovalino-Cordova AM , Fogliano V & Capuano E (2018) A closer look to cell structural barriers affecting starch digestibility in beans. Carbohydr Polym 181, 994–1002. 29254064 10.1016/j.carbpol.2017.11.050

[19] Fattore E , Botta F & Bosetti C (2021) Effect of fructose instead of glucose or sucrose on cardiometabolic markers: a systematic review and metaanalysis of isoenergetic intervention trials. Nutr Rev 79, 209–226. 33029629 10.1093/nutrit/nuaa077

[20] Ranawana V , Leow MKS & Henry CJK (2014) Mastication effects on the glycaemic index: impact on variability and practical implications. Eur J Clin Nutr 68, 137–139. 24219890 10.1038/ejcn.2013.231

[21] Tan VMH , Ooi DSQ , Kapur J , et al. (2016) The role of digestive factors in determining glycemic response in a multiethnic Asian population. Eur J Nutr 55, 1573–1581. 26160548 10.1007/s00394-015-0976-0

[22] Ranawana V , Monro JA , Mishra S , et al. (2010) Degree of particle size breakdown during mastication may be a possible cause of interindividual glycemic variability. Nutr Res 30, 246–254. 20534327 10.1016/j.nutres.2010.02.004

[23] Atkinson FS , Hancock D , Petocz P , et al. (2018) The physiologic and phenotypic significance of variation in human amylase gene copy number. Am J Clin Nutr 108, 737–748. 30239565 10.1093/ajcn/nqy164

