# Supplementary material for: Glycaemic potency reduction by coarse grain structure in breads is largely eliminated during normal ingestion
Source: Br J Nutr. 2021 Jul 5;127(10):1497–505. doi: 10.1017/S000711452100252X (PMC9044219; doi:10.1017/S000711452100252X)
Supplement: Supplementary file 1 [file S000711452100252Xsup001.docx]

**Supplementary material**

Grain Milling

The hard purple wheat was supplied de-hulled and uncooked or otherwise processed. The soybeans were passed through a Kenwood Grain Mill AT941A to coarsely grind the seed and facilitate separation of the seed coat. The seed coat was blown from the partially ground endosperm using a heat gun set to the coolest setting. The purple wheat and soy particles were then milled using a Kenwood Grain Mill AT941A with variable feed rate and two fluted rollers operating at different speeds. The gaps between the rollers was adjusted by screws to mill the gain to the desired particle sizes. The milled grain was sieved (> 2.8, aperture) using a mechanical shaker (Model RX-6-1, W.S Tyler, 8570 Tyler Blvd., Mentor, OH 44060, USA) for 5 minutes, the sample fraction remaining on the sieves was collected to make the breads.

Bread Baking

The kibbled-grain breads and reference white breads were prepared in the The New Zealand Institute of Plant and Food Research Limited, food safe laboratory. The standard white bread (control) was formulated with white flour plus (g/100g of wheat flour basis) 1.9g salt, 1.9g sugar, 3.8g gluten, 3.2g yeast, 0.6g dough improver, 3.4g oil to which water was added to 73.6% of the final dough. Variations to make the kibbled-grain breads are described below. The yeast was dissolved in warm water (33±2 °C) and left standing for 10 min. The remaining dry ingredients were placed in a bowl and mixed KitchenAid® Mixer (Model 5KSM150PS, KitchenAid, USA) fitted with a dough hook at speed level 2. The fresh yeast suspension, oil and water were then added, and mixing was continued for 12 min. The resulting dough was then divided into 400 g pieces, and shaped into loaves and placed into 27.6 x 14 x 6.5 cm silicone loaf pan and sealed tightly with metal foil wraps allowing space for leavening of the bread. The loaves were then placed in a standard bread proofer (Irinox MF 70.1) (30 °C, 100% humidity) for 60 min for the initial proofing. After 60 min, the dough was again kneaded in the mixer (KitchenAid®) for 5 min. The dough was again shaped into loaves and placed into the loaf tins and tightly covered with metal foil allowing space for the leavening of the breads. The breads were finally proofed at 30 °C, 100%RH (Irinox MF 70.1) for 60 min, after which they were baked in a conventional oven (Zanussi Professional) at 215 °C with the air vent shut to prevent moisture loss until the internal temperature of the loaves reached 90° C (Traceable® Food Thermometer). After baking, the loaves were cooled for 1 h until the internal temperature of the bread reached 30 °C, and then cut into slices, packed and labelled in polyethylene ziplock bags and stored at -20°C until analysis.

For the breads containing 75% of kibbled-grain, the grain particles were hydrated by soaking for 16 hr at 20° C after which they were drained and blotted dry. The white bread matrix was prepared as above and kneaded for 10 min. After 10 min the appropriate quantity of the blotted grains **Table 1** were added to the white dough mixture and kneaded for a further 2 mins. The dough was divided into 800 g pieces, shaped into loaves, and proofed and baked as for the white bread. The dough weight of kibbled-grain breads was double that of the white bread to give loaves of similar dimensions to the white bread when baked. After baking, the loaves were cooled, packed and stored as for the white breads. All the test breads were baked, sliced, packed and frozen before the human study.
